# *Mycobacterium chimaera* infections in cardiothoracic surgery patients exposed to heating and cooling devices despite infection control measures

**DOI:** 10.1017/ash.2023.230

**Published:** 2023-09-29

**Authors:** Jensie Burton, Yosra Alkabab, Susan Dorman, Jeremy D. Moore, Danny Nixon, Cassandra Salgado, Scott Curry

## Abstract

**Background:** LivaNova 3T heating and cooling devices (HCDs) have been associated with *Mycobacterium chimaera*, a *Mycobacterium avium*-intracellulare (MAIC) species, infections after cardiothoracic surgery. We describe our outbreak, which persisted despite escalating infection control measures. **Methods:** We identified patients with a positive MAIC culture following cardiothoracic surgery from January 2015 to the present at our institution. We classified these as “definite,” “possible,” or “operating room contamination” cases based on positive cultures from sterile sites, airway, or surgical specimens without evidence of infection. To identify patient or surgery characteristics associated with risk for MAIC infection, we conducted a case–control study comparing definite cases to randomly selected unmatched controls of patients over the same period without a positive MAIC culture after cardiothoracic surgery. **Results:** We identified 26 patients with a positive MAIC culture after cardiothoracic surgery: 13 definite, 9 possible, and 4 contamination cases. Among definite cases, the most common surgeries were valve replacements and left ventricular assist devices (5 cases each). The mean time from cardiothoracic surgery to diagnosis was 525 days. Overall, 10 (77%) cases occurred after exposure to our oldest HCDs (manufactured in 2013 or earlier). To date, 16 (62%) have undergone or are undergoing treatment for MAIC infection, and 4 (15%) have died due to NTM infection or complications. Compared to 47 controls, definite cases were associated with chronic kidney disease, implants, procedure type, use of cardiopulmonary bypass, and HCD age. Cases were not associated with time on bypass, time in the operating room, or other comorbid conditions (Table). All cases occurred despite enhanced disinfection and reorienting the HCD within the operating room, according to manufacturer recommendations. Moreover, 18 cases, including 7 definite cases, occurred after most HCDs were either deep cleaned or upgraded by the manufacturer. Also, 5 cases, including 3 possible cases and 2 contamination cases, occurred after physical separation of the HCD from the operating room. In August 2022, we purchased a fleet of glycol-cooled HCDs, and we have not identified additional MAIC cases since their deployment (Fig.). **Conclusions:** MAIC infections after cardiothoracic surgery were associated with procedure type, especially implants, use of cardiopulmonary bypass, and HCD age. Contrary to prior reports, neither operative nor CPB time was associated with MAIC infection after cardiothoracic surgery. The outbreak persisted despite disinfection and/or deep cleaning and reorienting HCDs within the operating room; some possible and contamination cases occurred even after moving HCDs outside the operating room. Thus, HCD water contamination events in the operating room (eg, spills from HCD tubing) may be a route of exposure, and different infection prevention measures are needed.

**Figure:**
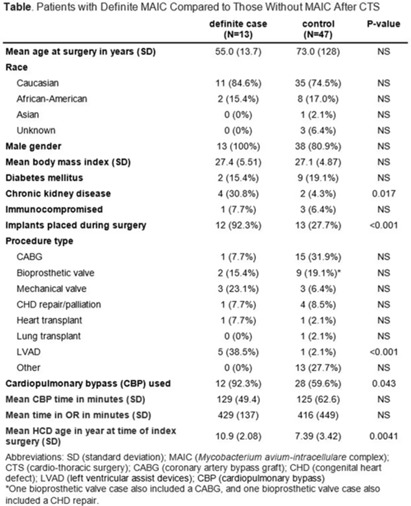

**Figure:**
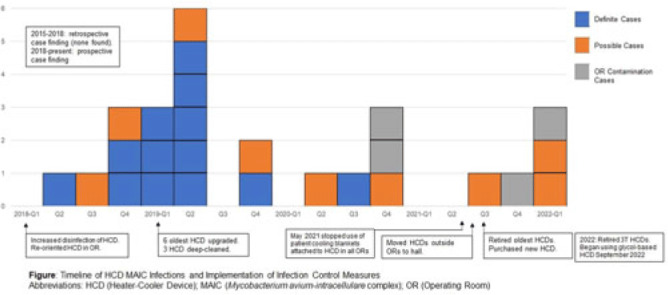

**Disclosure:** None

